# Electrical and Structural Adaption of Athlete’s Heart and the Impact on Training and Recovery Management in Professional Basketball Players: A Retrospective Observational Study

**DOI:** 10.3389/fphys.2022.739753

**Published:** 2022-02-11

**Authors:** Paul Zimmermann, Othmar Moser, Frank Edelmann, Volker Schöffl, Max L. Eckstein, Martin Braun

**Affiliations:** ^1^Department of Cardiology, Klinikum Bamberg, Bamberg, Germany; ^2^Interdisciplinary Center of Sportsmedicine Bamberg, Klinikum Bamberg, Bamberg, Germany; ^3^Division of Exercise Physiology and Metabolism, Department of Sport Science, University of Bayreuth, Bayreuth, Germany; ^4^Interdisciplinary Metabolic Medicine Research Group, Division of Endocrinology and Diabetology, Medical University of Graz, Graz, Austria; ^5^Department of Internal Medicine and Cardiology, CVK, Charité University Medicine Berlin, Berlin, Germany; ^6^German Centre for Cardiovascular Research, Partner Site Berlin, Berlin, Germany; ^7^Department of Traumatology and Orthopaedics, Klinikum Bamberg, Bamberg, Germany

**Keywords:** cardiac remodeling, training management, recovery, echocardiogaphy, cardiopulmonary exercise test

## Abstract

**Introduction:**

We analyzed data of 27 professional basketball players to prove cardiac remodeling referring echocardiographic parameters, cardiopulmonary exercise testing (CPET), and 12-lead electrocardiogram (ECG) analyses. The aim of our study was to present different characteristics in the athletes, on the one hand signs of a high vagal tone in the 12-lead ECG as criteria of early repolarization (ER), furthermore echocardiographic remodeling parameters and finally the performance in CPET. Therefore, we divided the cohort into a group with signs of ER pattern in the 12-lead ECG and without these criteria and presented the differences in detail.

**Materials and Methods:**

This was a single-center, retrospective study performed in 27 professional basketball players (age: 26.5 ± 7.5 years, male: 27, height: 197.2 ± 12 cm, weight: 100 ± 17 kg, BMI: 25.7 ± 3.4 kg/m^2^). All participants underwent a sports medicine checkup, ECG analysis, transthoracic echocardiographic examination, and a CPET on a cycle ergometer between 2015 and 2019 during their pre-season preparation time. All individuals were healthy people without cardiological advance anamnesis. After assessment, two groups were built based on electrocardiographic criteria of ER pattern and a group without these criteria and compared against each other for parameters of echocardiographic assessment, CPET, and 12-lead ECG analysis. Data were analyzed with Minitab statistic program (Minitab Inc., State College, PA, United States) and Graph Pad Prism 8.2.1 (279; Graph Pad Software, San Diego, CA, United States) using ANOVA testing with *post-hoc* testing and unpaired t-testing (*p* ≤ 0.05).

Retrospectively additional information was collected referring to the management of training sessions, recovery time, and nutrition by interviewing the athletic training staff in order to understand the principles for individual athlete’s training management and physiological and cardiopulmonary interactions.

**Results:**

Comparing professional basketball players with ER pattern to those with no ER pattern, significant differences were found for CPET, echocardiographic, and ECG analysis (*p* < 0.05). Absolute and relativized peak oxygen uptake (VO_2 peak_; ER 4120 ± 750 ml/min (39 ± 5.4 ml/kg/min) vs. non-ER 3556 ± 393 ml/min (37.2 ± 5.3 ml/kg/min), *p* = 0.018) and maximum workload during CPET (ER 310 ± 51.5 Watt (2.94 ± 0.35 W/kg) vs. non-ER 271 ± 32 Watt (2.85 ± 0.49 W/kg), *p* = 0.026) was higher in athletes with an ER pattern. Furthermore, ER pattern athletes showed a higher enddiastolic left ventricular diameter (LVedd; ER 58.3 ± 7.9 mm vs. non-ER 53.6 ± 3.6 mm, *p* = 0.048) and a significantly enlarged left atrial (LA) endsystolic diameter (ER 23.33 ± 2.71 mm vs. non-ER 20.47 ± 2.29 mm, *p* = 0.006) as well as a significantly enlarged right atrial (RA) endsystolic diameter (ER 23.42 ± 2.15 mm vs. non-ER 20.93 ± 3.28 mm, *p* = 0.033). Significant differences between the two compared groups could be revealed for left ventricular mass Index (LVMI gr/m^2^; LVMI ER 113 gr/m^2^ ± 17.5 vs. LVMI non-ER 91.3 gr/m^2^ ± 15.1, *p* = 0.002), but no significant differences for the relative wall thickness were found (RWT; RWT ER 0.49 ± 0.11 vs. RWT non-ER 0.38 ± 0.06, *p* = 0.614).

**Conclusion:**

Professional basketball players with criteria of ER pattern showed different results in CPET and cardiac remodeling as athletes with no ER pattern. These findings should encourage the athletic training staff to emphasize the quality of an individual training schedule for each athlete based on the cardiopulmonary pre-season sport medicine checkup. Nevertheless, echocardiographic findings, ER pattern, and performance in CPET have to be interpreted referring the sport-specific and athlete’s ethnical background.

## Introduction

Physical exercise has been shown to reduce all-cause mortality, atherosclerosis, type 2 diabetes, and cancer (Makura et al., 2013; Ried-Larsen et al., 2015). Although regular physical exercise is beneficial for reducing cardiovascular morbidity and mortality, different exercise activities cause anatomic adaptations in athletes’ heart structure. Those are associated with atrial fibrillation (AF), alterations in the autonomic nervous system inducing early repolarization (ER), chronic systemic inflammation, and fibrosis (Guasch et al., 2013; Wilhelm, 2014; Harada et al., 2015). AF is a commonly detected abnormality of the heart rhythm found in the general population as well as in athletes (Guasch et al., 2013; Wilhelm, 2014). Several studies have reported a high prevalence of AF in athletes mainly dependent on exercise intensity when compared against the general population (Aizer et al., 2009; Sorokin et al., 2011). Various potential trigger mechanisms have been discussed (Müssigbrodt et al., 2010; Calvo et al., 2012; Mascia et al., 2013; Fragakis et al., 2014; Sanchis-Gomar et al., 2017), summarized as the so-called “PAFIYAMA” (“paroxysmal AF in young and middle-aged athletes”) representing a finding in middle-aged athletes, especially endurance athletes. In these endurance athletes, an ER pattern is often detected in the ECG as a benign finding which is associated with training and does not require further evaluation (Drezner et al., 2017) that might potentially be a surrogate parameter for an increased vagal tone (Haydar et al., 2000; Maron and Pelliccia, 2006; Mont et al., 2009; Reinhard et al., 2019). The prevalence of ER as a common finding in healthy population is estimated to be 2–44% (Drezner et al., 2017). In athletes, the ER pattern is reported in up to 45% in Caucasian athletes and 63–91% in black athletes. It seems to be affected by exercise frequency and peak fitness levels (Drezner et al., 2017). The ER pattern, which was initially described in 1936, has been estimated as a benign ECG finding for more than 50 years (Shipley and Hallaran, 1936). However, this view changed in 2008, when Haïssaguerre et al. reported their findings (Haïssaguerre et al., 2008). Among patients with history of idiopathic ventricular fibrillation (VF) or sudden cardiac arrest, there are reports of increased prevalence of ER in case–control studies (Haïssaguerre et al., 2008; Rosso et al., 2008). Large population-based studies confirmed an association between increased cardiac risk for all-cause mortality and the prevalence of ER pattern in middle-aged subjects (Tikkanen et al., 2009; Sinner et al., 2010). The association between the occurrence of heart rhythm abnormalities, such as the occurrence of AF and the peak performance during cardiopulmonary exercise testing (CPET), has been reported in different groups of athletes, mainly focused on endurance sports (Maron and Pelliccia, 2006; Aizer et al., 2009; Sorokin et al., 2011; Guasch et al., 2013; Wilhelm, 2014). Therefore, we analyzed data of 27 male professional basketball players to detect cardiac remodeling referring echocardiographic parameters, CPET and ECG analysis, focused on this moderate dynamic sport category, which combines strength and endurance components in the aerobic and anaerobic system.

The primary aim of our retrospective study was to evaluate functional and structural cardiac remodeling by echocardiographic and CPET analyses in professional basketball players. We hypothesized to find different effects of basketball training on cardiovascular function, that is, cardiac functional and structural remodeling as well as different outcomes in CPET in athletes with ER pattern and players without ER pattern. In this context, the ER pattern is a controversially discussed topic and is associated as a benign finding in athlete’s ECG interpretation considering ethnicity on the one hand as well as a potentially crucial cause for future heart rhythm abnormalities in athletes on the other hand. Therefore, ER pattern analysis might contribute to individualized multidisciplinary exercise plans in the future and potentially play an important role for the future estimation of athlete’s performance.

## Materials and Methods

This was a single-center retrospective observational study performed in 27 male professional basket players during their annual pre-season sport medicine checkup.

### Eligibility Criteria

Twenty-seven professional basketball players were examined during 2015–2019. The athletes were in the first pre-season preparation phase, which is mainly characterized by high-volume with low intensity training. All participants underwent a sports medicine checkup including physical, orthopedic and cardiovascular examination, 12-lead ECGs, transthoracic echocardiographic examination, and a CPET on a bicycle ergometer as part of their preparticipation screening for the national basketball league in Germany. In this monocentric study, the transthoracic echocardiographic analyses were performed by the same physician as well as the CPET interpretation. Participants were not included in the study if they had any diagnosis of a relevant cardiac comorbidity or no pre-cardiological anamnesis was conducted.

### Visits

As part of the sports medicine checkup, we performed 12-lead ECGs in lying position with 50 mm/s (CardioSoft V6.73, GE Medical Systems, Germany). We performed an echocardiographic functional and morphological assessment using a commercially available echocardiographic system Phillips EPIQ 7 device with an X5-1 aMatrix-array transducer (Phillips Healthcare, Eindhoven, Netherlands), following a standardized protocol (Evangelista et al., 2008). The images were stored and analyzed digitally; for measurements, sequences of at least three heart beats were stored and analyzed. The left ventricular systolic ejection fraction (LV-EF) was estimated and calculated by the biplane Simpson rule based on the apical four- and the apical two-chamber view and the enddiastolic diameter of the left ventricle (LVedd) was measured in the parasternal long axis. An endsystolic planimetry of both atrial sizes (right and left atrium, RA and LA) in the apical four-chamber view was performed as well as an estimation of the right ventricular (RV) systolic function using the TAPSE (Tricuspid annular systolic excursion) in the apical four-chamber view. Based on the two-dimensional echocardiographic measurements the left ventricular mass index (LV Mass index, LVMI) was calculated with a validated method (Hashem et al., 2015), the relative wall thickness (RWT) of the left ventricle (LV) was calculated as (2× posterior wall thickness)/LVedd (Hashem et al., 2015). For assessment of the LV diastolic function, we measured the pulse-wave Doppler in the apical four-chamber view referring the peak early filling (E wave) and late diastolic filling (A wave) velocities. A tissue Doppler imaging of the lateral mitral anulus in the apical four-chamber view was performed (peak early velocity E′) (Lang et al., 2006; Evangelista et al., 2008).

The ER pattern was defined according to the early repolarization standard of the American Heart Association (AHA), referring to the classic definition of early repolarization and the new definitions of early repolarization by the American College of Cardiology Foundation (2015) (Macfarlane et al., 2015). Furthermore, the ECG interpretation was performed according to the 2017 International Recommendations for ECG Interpretation in Athletes by Sharma et al. (2017). Therefore, the athletes’ ECG was evaluated referring ER based on the following criteria: concave elevation of the QRS-ST junction (J-point) by ≥0.1 mV often associated with late QRS slurring or notching (J-wave, defined as a deflection after the QRS complex as a small secondary R wave or a late delta wave), affecting the inferior and/or lateral ECG leads (Macfarlane et al., 2015; Aagaard et al., 2016; Sharma et al., 2017). In our collective, we defined two groups: athletes presenting with criteria of ER pattern and no criteria of ER pattern in their 12-lead ECG.

The exercise testing was conducted in accordance to the recommendations of the AHA (Fletcher et al., 2001). The CPET stepwise protocol started with a workload of 80 Watts, and the workload was increased by 40 Watts every 3 min until volitional exhaustion. We continuously recorded all data from the CPET measurements (heart rate using a 12-lead ECG), blood pressure measurement at each stage and up to 3 min after the end of exercise. The mean duration of the maximal CPET was 27 ± 4 min in the ER pattern group and 26 ± 4 min in the non-ER pattern athlete’s cohort. The peak criteria for CPET were defined as following: Reaching 85% of the maximum predicted heart rate (220 bpm minus age in years), respiratory exchange rate (RER) 1.15 at peak performance, time of CPET duration, evaluation of the athlete’s exertion level analogue Borg scale, evaluation of the peri- and post-exercise lactate level measured by capillary blood analysis from the earlobe, and the VO_2 max_ leveling off in the CPET analyses. To define a satisfied athlete’s maximal CPET effort in our cohort, three of the above-mentioned criteria were taken into consideration.

Additional information about the management of training sessions was collected retrospectively. In particular, detailed information about the conditioning program in the pre-season as well as during the season, the recovery time, and nutrition were evaluated by interviewing the athletic training staff and basketball players in order to understand the principles for individual athlete’s training as strength training (ST), movement training skills, and competitive basketball agility training.

These aspects were collected to emphasize the importance of nutritional requirements and resting periods, which play a fundamental role in the recovery of the basketball players during the competition season, based on strength, athletic, and competitive training as well as recovery strategy and resting (Ott and Santos, 2020). In several studies, these aspects were highlighted to provide appropriate training strategies that resemble match-play (Petway et al., 2020).

### Statistical Analyses

Data were analyzed with Graph Pad Prism 8.2.1(279) (Graph Pad Software, San Diego, CA, United States) and Minitab statistic program (Minitab Inc., State College, PA, United States). Our data were not normally distributed; therefore, we evaluated our numerical data group comparison for the 27 professional basketball players with ER pattern and athletes with no ER pattern using ANOVA testing with post-hoc testing and unpaired *t*-testing (*p* ≤ 0.05). Parametric data are presented as mean ± SD. Furthermore, a simple linear regression analysis was performed in order to determine the relation between pairs of continuous variables and a multivariate and stepwise linear regression analysis was performed to identify independent determinants of peak oxygen uptake (VO_2 peak_) during CPET.

### Ethical Consideration

The local ethics committee of the University of Nurnberg-Erlangen approved the study protocol (265_20 Bc). The study was conducted in conformity with the declaration of Helsinki and Good Clinical Practice (Harriss et al., 2019). Before any trial-related activities, potential participants were informed about the study protocol and participants gave their written informed consent.

## Results

Athletes had a mean age of 26.5 ± 7.5 years, male: 27, height: 197.2 ± 12 cm, weight: 100 ± 17 kg, BMI: 25.7 ± 3.4 kg/m^2^. Referring ethnicity, we analyzed the data of male black American (*n* = 13) and male Caucasian American and European athletes (*n* = 14).

In the ER pattern group, five basketball players of black origin and seven professional athletes of white Caucasian and American origin were included. In the non-ER pattern, cohort eight black American players and seven Caucasian and American white origin players were included. Participants had no sign of a pathology as examined by a study physician.

In 12-lead ECGs evaluation, five athletes (18.5%) showed a sinus bradycardia at rest and 12 athletes (44.4%, five basketball players of black origin and seven athletes of white Caucasian and American origin) had an ER pattern according to our defined ER standard (Macfarlane et al., 2015; Aagaard et al., 2016; Sharma et al., 2017). There were no statistical differences concerning the QTc intervals comparing the athletes with and without ER pattern (ER QTc 404 ± 27 ms vs. non-ER 409 ± 15 ms, *p* = 0.55) and no statistical differences between the heart rate at rest (ER 66 ± 10 bpm vs. non-ER 72 ± 12 bpm, *p* = 0.14) and the maximum heart rate at the peak workload testing (ER 172 bpm ± 10 vs. non-ER 165 ± 15 bpm, *p* = 0.19) between the two different groups could be detected. Both groups reached at the peak workload a similar level of the oxygen pulse at a respiratory exchange rate reaching RER > 1.15 (ER 23.9 ± 4.1 ml vs. non-ER 21.6 ± 2.5 ml, *p* = 0.08).

In the echocardiographic examination, athletes showed a normal LV-EF (mean LV-EF 60 ± 5 percent by the biplane Simpson rule). No signs of left ventricular hypertrophy with an average left ventricular wall thickness (IVSd mean 10.6 mm) could be detected. No athlete showed a severe left ventricular hypertrophy or another relevant cardiomyopathy [CMP; i.e., for example a non-compaction cardiomyopathy (NC-CMP) or Hypertrophic obstructive cardiomyopathy (HOCMP)].

**Figure 1:**
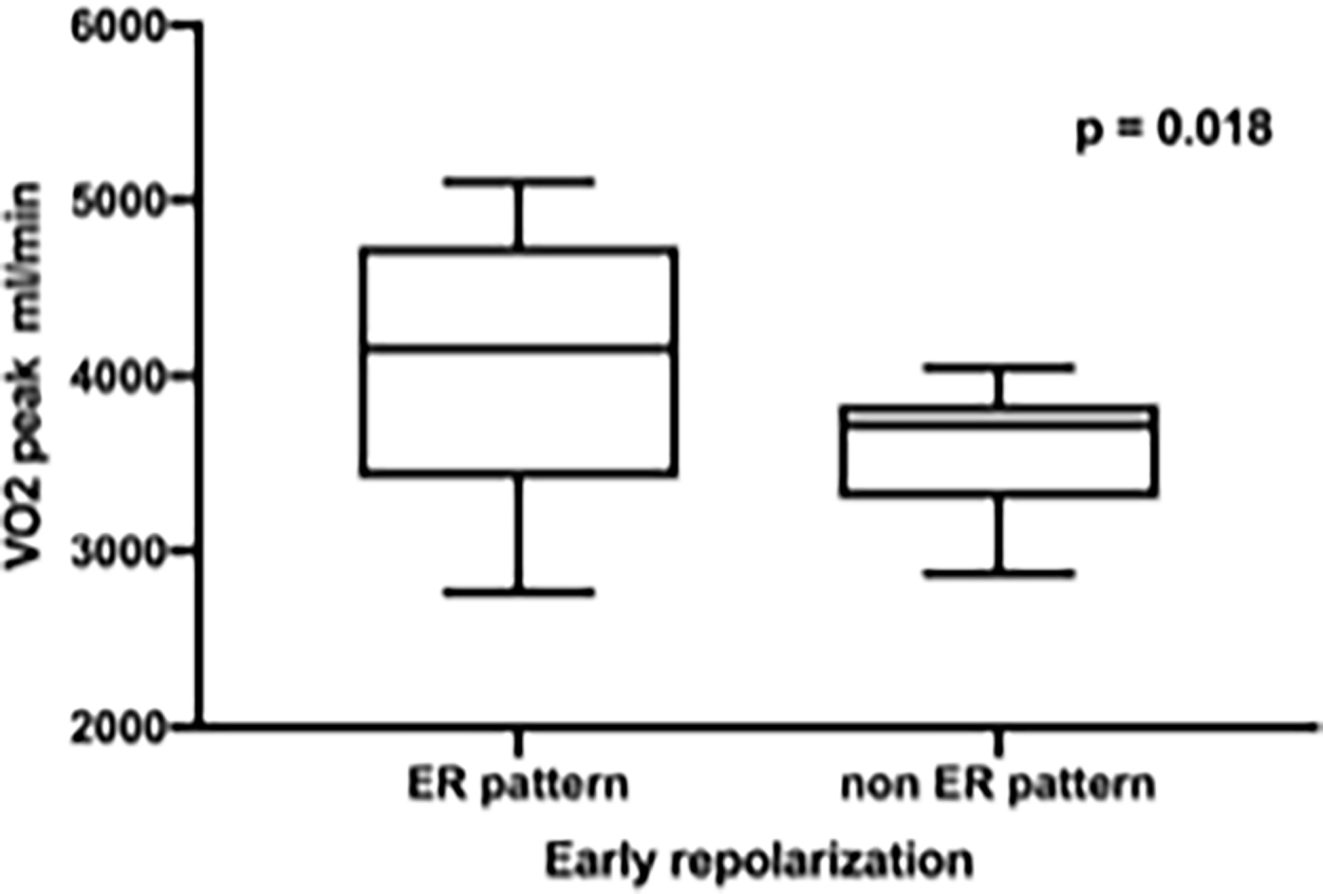
Significant higher peak performance (VO_2 peak_) in professional basketball players with early repolarization (ER) compared to non-ER pattern.

**Figure 2:**
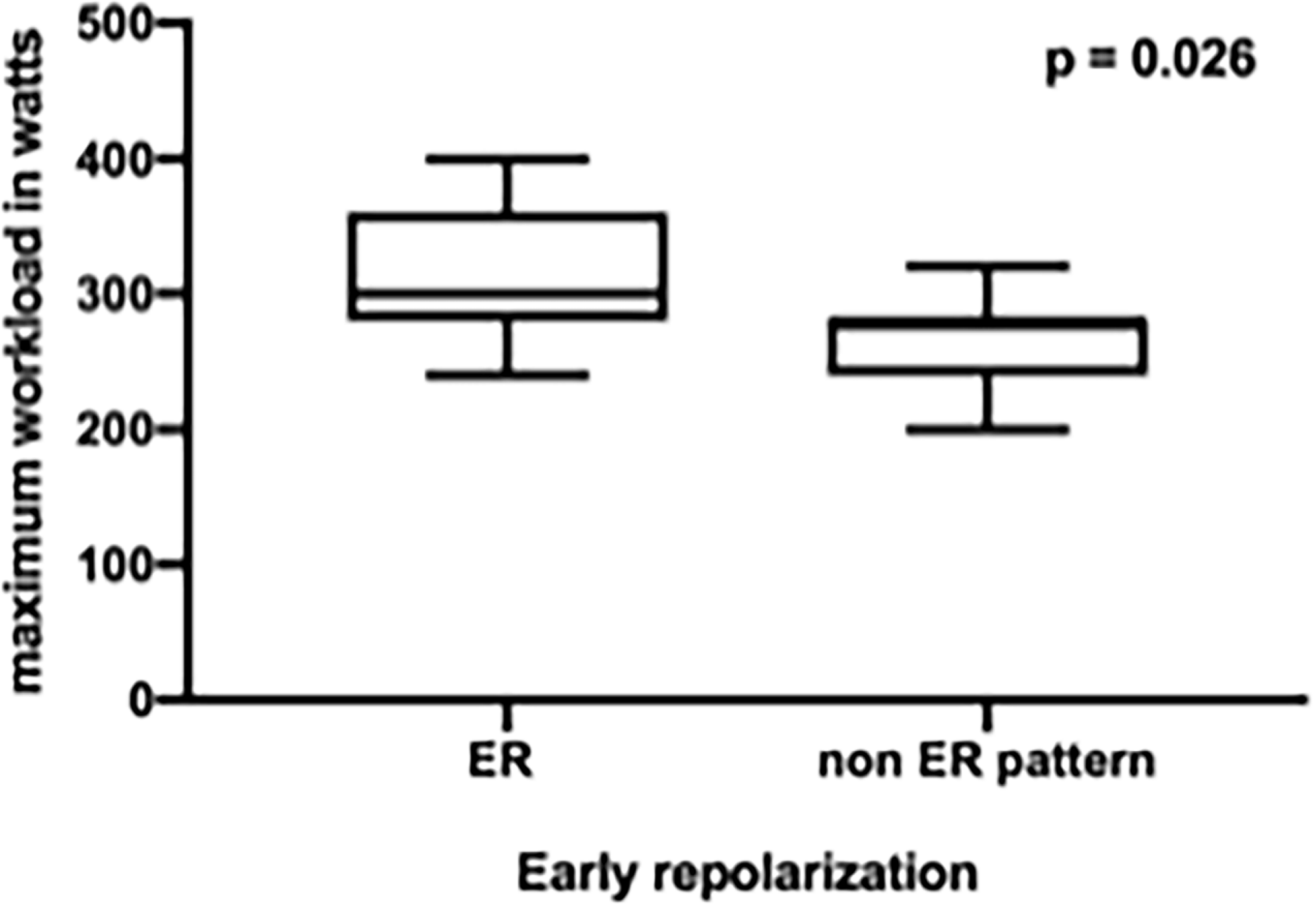
Increased maximum workload in professional basketball players with early repolarization (ER) compared to non-ER pattern.

Comparing the professional basketball players with ER pattern to those without ER pattern, we found significant differences during the CPET: basketball players with an ER pattern showed a higher peak performance (VO_2 peak_) and weight-specific oxygen uptake [ER 4120 ± 750 ml/min (39 ± 5.4 ml/kg/min) vs. non-ER 3556 ± 393 ml/min (37.2 ± 5.3 ml/kg/min), *p* = 0.018, Figure 1] and an increased maximum workload during CPET [310 ± 51.5 Watt (2.94 ± 0.35 W/kg) vs. 271 ± 32 Watt (2.85 ± 0.49 W/kg), *p* = 0.026, Figure 2].

**Figure 3:**
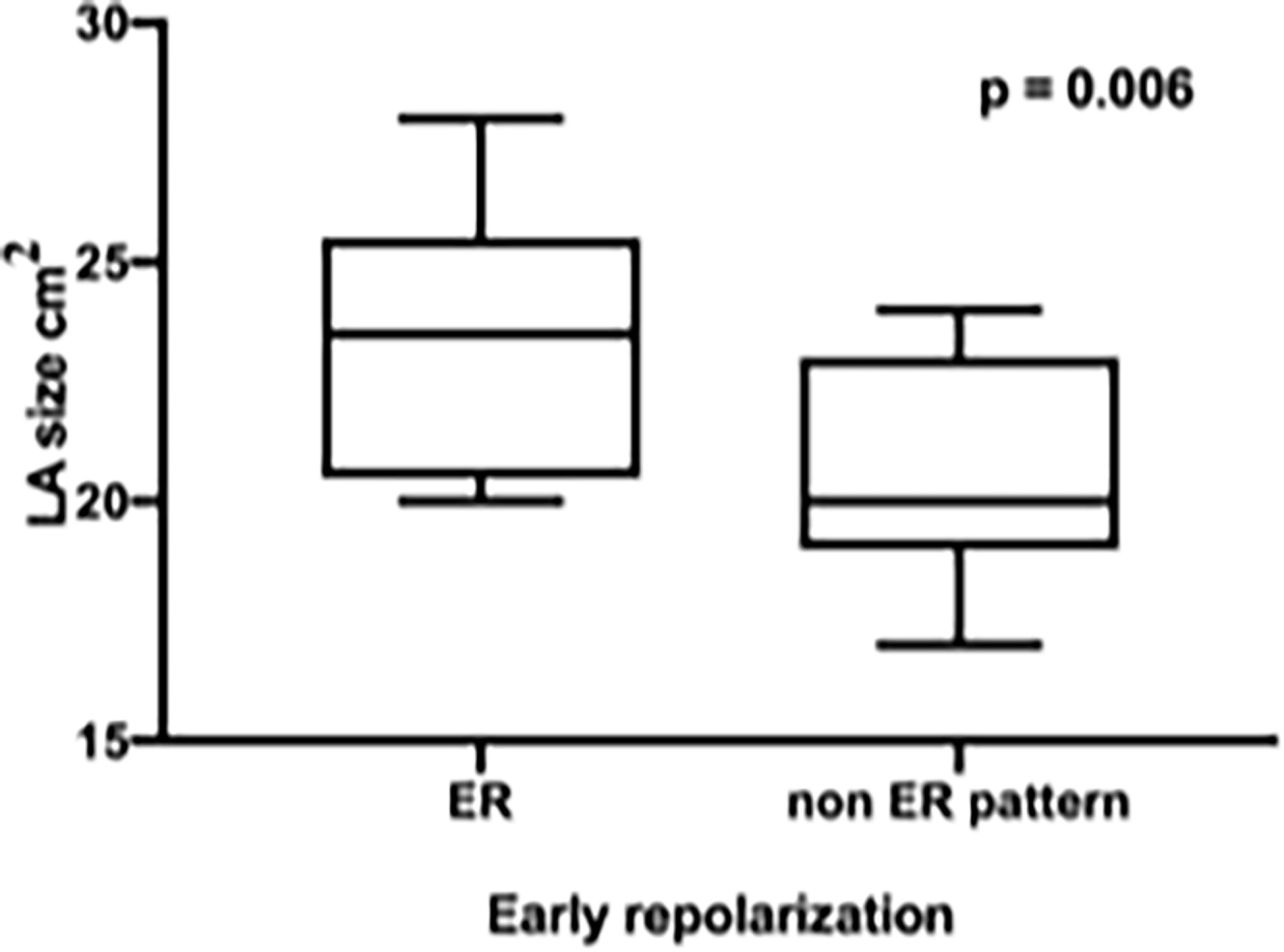
Significantly enlarged LA endsystolic diameter in professional basketball players with early repolarization (ER) compared to non-ER pattern.

**Figure 4:**
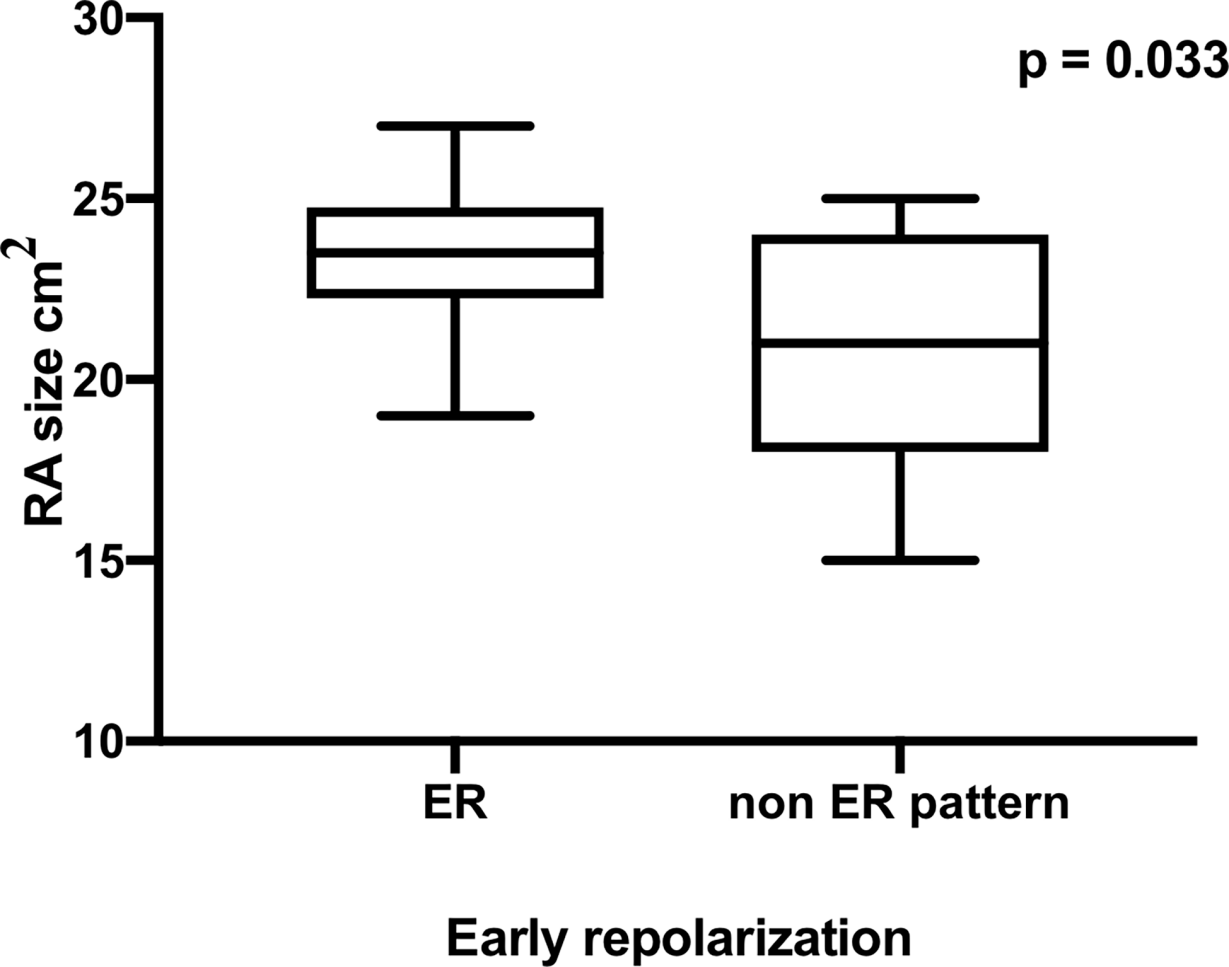
Significantly enlarged RA endsystolic diameter in professional basketball players with early repolarization (ER) compared to non-ER pattern.

Furthermore, the ER pattern athletes showed a significant higher LVedd (ER 58.3 ± 7.9 mm vs. non-ER 53.6 ± 3.6 mm, *p* = 0.048) and a significantly enlarged LA endsystolic diameter (ER 23.33 ± 2.71 mm vs. non-ER 20.47 ± 2.29 mm, *p* = 0.006, Figure 3) as well as a significantly enlarged RA endsystolic diameter (ER 23.42 ± 2.15 mm vs. non-ER 20.93 ± 3.28 mm, *p* = 0.033, Figure 4).

Significant differences between the two compared groups could be revealed for left ventricular mass Index (LVMI gr/m^2^, LVMI ER 113 gr/m^2^ ± 17.5 vs. LVMI non-ER 91.3 gr/m^2^ ± 15.1, *p* = 0.002), but no significant differences for RWT (RWT ER 0.49 ± 0.11 vs. RWT non-ER 0.38 ± 0.06, *p* = 0.641). Analyzing the ER athletes for ethnic origin due to LVMI and RWT no significant differences could be proven for black American players in comparison to white Caucasian and American players (LVMI ER black 115.8 gr/m^2^ ± 17.9 vs. ER white 111 gr/m^2^ ± 18.3, *p* = 0.66 and RWT ER black 0.45 ± 0.11 vs. RWT white 0.36 ± 0.04, *p* = 0.14).

While comparing the TAPSE as a parameter for the systolic function of the RV and the E/E′ ratio as criteria for LV diastolic function, there was no significant difference between basketball players with ER pattern or without ER pattern (ER TAPSE 28.3 ± 5.7 mm vs. non-ER TAPSE 27.7 ± 6.6 mm, *p* = 0.81 and ER E/E′ ratio 5.92 ± 0.9 vs. non-ER E/E′ ratio 5.93 ± 1.4, *p* = 0.97).

**Figure 5:**
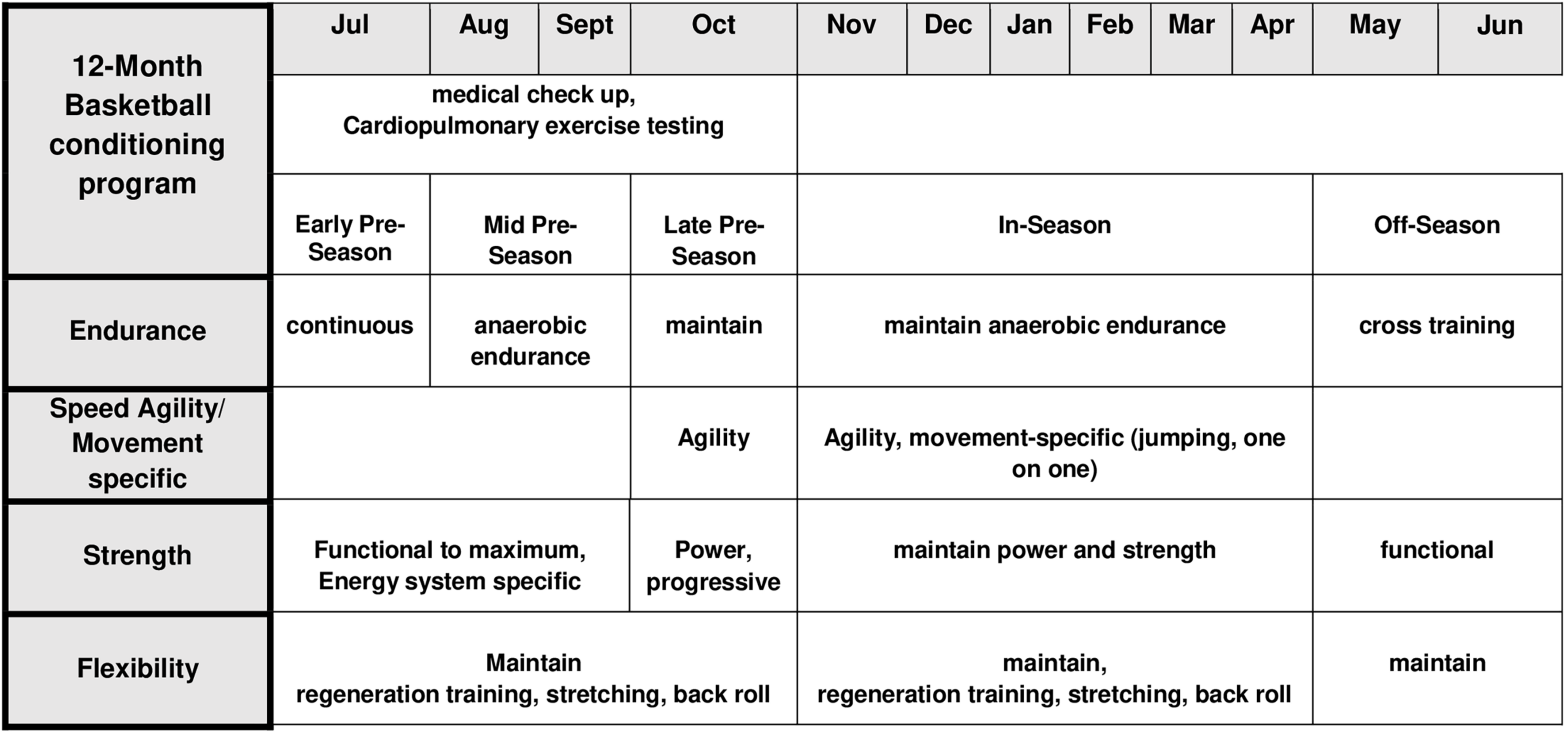
Example of a 12—month basketball training program.

The conditioning program in the pre-season including energy-specific ST, movement-specific and competitive training as well as progressive ST was the same for all athletes, just mandatory individual athletic training was added to improve muscle disbalances. The endurance training (ET) program during the season was based on short and high intensity training between the competition days and movement-specific training with jumping, defensive slides and competitive aspects as training with teammates or time trials (Figure 5). The ET was performed in the pre-season conditioning program, whereas opponent basketball analysis, competition strategy conferences and maintaining power and ST were focused In-season time. Physiotherapy, sufficient hydration status, mineral nutrients, and at least 8 h of sleep each night were important during the whole year to minimize fatigue and injury risk and were offered each athlete by the athletic and coaching staff.

## Discussion

In our retrospective observational study, we found that professional male basketball players, which showed a higher peak performance (VO_2 peak_) in CPET, had more often an ER pattern in the 12-lead ECG. This finding is a common phenomenon in athletes, which is reported up to 45% in Caucasian athletes and 63–91% in black athletes and seems to be affected by exercise training (Drezner et al., 2017). This improved functional capacity might cause an increased vagal tone; furthermore, one the other hand, there is some evidence that the accumulation of life time training hours and participation in competitive sports increase the risk for AF and ER in endurance athletes (Wilhelm, 2014) as well as the ethnical background seems to play an important role in this context as discussed hereinafter.

Some studies reported that even veteran male endurance athletes showed an increased rate of AF, and left atrial remodeling, but no differences in LV-EF or significant differences in diastolic function (Wilhelm et al., 2011). Our results, which are reporting these cardiac adaptations and specific structural remodeling for the first time in professional male basketball players, are supported by another study, which revealed in elite athletes associations in the notching ER subtype pattern with LA enlargement, but could not prove distinct echocardiographic alterations and significant overall differences in CPET in ER athletes; in this study, athletes were included from different types of sport, that is, low to high static and low to high intensity sports (Reinhard et al., 2019). In general, ER pattern is known as a surrogate parameter for increased vagal tone as reported before (Haydar et al., 2000; Barbosa et al., 2008), and according to the results reported in soccer players (Stumpf et al., 2016), basketball players with ER pattern showed a higher peak performance (VO_2 peak_) in CPET compared to non-ER pattern professional basketball players. These circumstances have to be taken into consideration while evaluating patterns of ER and structural as well as functional cardiac remodeling in competitive athletes. Up to now, professional athlete’s ER pattern without clinical markers of pathology and presented in isolation should be considered benign variants in athletes. Further studies in this scientific research area are necessary to elucidate the prognostic value of ER pattern analysis in professional athletes (Drezner et al., 2017).

No statistically differences could be proven for the analyzed ECG parameters in our study cohort. There were no statistical differences concerning the acquired data at rest, as the QTc intervals and the heart rate at rest, but also no significant changes in the dynamic parameters, such as the maximum heart rate at the peak workload testing or the peak workload oxygen pulse.

In comparison to other athletes of different sports, we could not detect significant differences referring to the diastolic function, that is, E/A ratio and E/E′ ratio in our two different groups (Stumpf et al., 2016). Nevertheless, we tried to prove independent determinants of VO_2 peak_ in our cohort, but no significant coherences between the different echocardiographic and ECG data could be proven. The only fact we could reveal is that a low E/A ratio in ER pattern athletes seems to show an inverse correlation to peak performance (VO_2 peak_). Therefore, a secondary increased vagal tone might play a role or the specific cardiac remodeling—discussed in the further sports career, that is, biatrial enlargement because of high atrial filling pressure and sport-related fibrosis might contribute to this inverse correlation.

Regular participation in intensive sport competition due to discipline and intensity as well as age, sex, and ethnicity play an important role in developing electrical and structural alterations in athlete’s heart which might manifest on the 12 lead surface ECG. Especially in large male and athletes of black African or Afro-Caribbean origin, pathological ECG feature might be obtained which are difficult to be distinguished from cardiomyopathies or channelopathies (Prakash and Sharma, 2016). Black athletes with African or Afro-Caribbean (black) origin show a higher prevalence of atrial enlargement and left ventricular hypertrophy. Next to these structural cardiac components ST segment alterations, *T* wave inversions, and ER pattern seem to be more prevalent in black athletes compared to non-black athletes—other data suggest ER pattern in 34% black athletes versus 28% in white athletes (Junttila et al., 2011; Ozo and Sharma, 2020). Therefore, adolescent athletes with ER pattern exhibited greater RWT and LVMI, suggesting a tendency to left ventricular concentric geometry remodeling, as reported by Miragoli et al. (2019). Different studies reveled a greater percentage of black athletes to be associated with abnormal ECG findings, as ER pattern (Demola et al., 2019; Miragoli et al., 2019; Pambo and Scharhag, 2021). We are aware of these diverse results due to the ethnicity of our professional male basketball players and reviewing our results we have to interpret the data in a different context. Our ER athletes showed signs of structural left ventricular and right atrial remodeling, a tendency to higher LVMI as concentric geometry LV remodeling, and higher performance values in CPET. Nevertheless in the subgroup analysis of the ER athletes, no ethnicity differences could be revealed due to LVMI and RWT in our small descriptive cohort. Delineating between physiological left ventricular remodeling and HCM is essential to prevent sudden cardiac death as adolescent black athletes demonstrate a 5% increase in mean left ventricular wall thickness compared to white athletes of similar age (di Paolo et al., 2012). The cardiac MRI plays an important role in this context to determine cardiac adaption to exercise referring the ethnicity background, and the awareness of these ethnic variants will prevent unwarranted exclusion from competitive sports (Ozo and Sharma, 2020).

The CPET and cardiological results were recognized by the coaching staff and taken into consideration for scheduling individual training sessions. The understanding of the relationship between external physical training demands and internal physiological responses should contribute to an individual dose–response training load for optimum competition preparation and injury prevention. Basketball is a court-based team-sport that requires a broad range of different demands, such as physiological, mechanical, technical, and tactical in training and competition. In several studies, these aspects are highlighted to provide appropriate training strategies that resemble match-play (Petway et al., 2020). These sport-specific challenges require a detailed management of training sessions—especially referring the conditioning program in the pre-season as well as during the season, the recovery time, and nutrition. Therefore, different tests are established to investigate the training load and neuromuscular adaption in the preparation period as described in 2017 by Ferioli et al. (2018). Furthermore, there is some effort as described by Fox et al. to establish a monitoring system in basketball training to understand the external demands and internal response of each basketball player. This monitoring allows the training staff to get the athletes prepared adequately for competition as well as minimize the factors as fatigue and injury risk (Fox et al., 2017). Berkelmans et al. reported applications and recommendations for heart rate monitoring in basketball training in order to monitor exercise intensity, assessing fatigue status, and quantifying the internal training load (Berkelmans et al., 2018). Next to the mentioned training demands, nutrition plays an important role as it was proven by interviewing the athletic training staff and basketball players. The nutrition before, during, and after the game or in high intensity training sessions is essential for a quick recovery of the player and may differ substantially between professional basketball players (Szczepańska and Spałkowska, 2012; Ott and Santos, 2020).

Up to now in our retrospective study, the cardiopulmonary pre-season sport medicine evaluation, especially focused on ECG parameters as ER pattern as a common known sign in athletes, did not influence the training staff team to create an individual multidisciplinary approach for each athlete. All the mentioned key aspects above show that planning a professional basketball 12 Month training program is influenced by many different internal physiological and external factors and even by the athlete himself, so that a professional multidisciplinary approach is necessary. In the future, these detailed cardiological structural and functional remodeling findings which lack of involvement in the actual trainings schedules should be taken into consideration and might contribute as novel perspectives to improve more specifically each athlete’s individual recovery, trainings management, health, and game performance.

## Limitations

Our study has several limitations, as the relatively small number of professional basketball players included might mitigate the transferability to a larger population. In this context, our retrospective observational study might serve as a descriptive reporting of these interesting exercise physiological and cardiac remodeling findings and might highlight the necessity for further multidisciplinary approaches to investigate the athlete’s cardiovascular performance. Furthermore, we examined the athletes in the pre-season preparation and did not perform longer follow-up periods. On the one hand, the ER pattern might be a surrogate parameter for an increased vagal tone as a typical feature in athlete’s heart—on the other hand, there might be a higher risk for athletes with increased VO_2peak_ to develop an ER pattern during a long sports career with the consequence of the athletes’ atrial and left ventricular remodeling and for future heart rhythm abnormalities. Therefore, future scientific follow-up research is necessary. The ethnicity of our professional male basketball players must be taken into consideration interpreting the study results. Therefore, athlete’s cardiac adaption to exercise should be judged in reference to the ethnicity background and the awareness of these ethnic variants.

Our study refers to an important exercise physiology and cardiac remodeling area, while regarding the effects of basketball training on cardiovascular function and highlighting the different outcomes in CPET and cardiac remodeling in male basketball professionals of different ethnicity focusing ECG ER pattern. The main strength of our descriptive study is the limited literature in this area and our descriptive preliminary data might pave the road to further scientific effort in this field of cardiopulmonary pre-season sport medicine evaluation and importance of multidisciplinary approach to improve athlete’s health and game performance.

## Conclusion

In conclusion, professional male basketball players with criteria of ER pattern showed different results referring structural and functional echocardiographic remodeling as well as to athlete’s performance during CPET in comparison to basketball professionals without an ER pattern. In our moderate dynamic sport cohort, professional male Basketball players with an ER pattern showed a higher peak performance (VO_2peak_), and weight-specific oxygen uptake, and an increased maximum workload during CPET as well as left ventricular and biatrial cardiac remodeling. These findings should encourage the athletic training staff to emphasize the quality of an individual training schedule for each athlete based on the cardiopulmonary pre-season sport medicine checkup. Nevertheless, echocardiographic findings, ER pattern, and performance in CPET have to be interpreted referring the sport-specific and athlete’s ethnical background.

## Data Availability Statement

The raw data supporting the conclusions of this article will be made available by the authors, without undue reservation.

## Ethics Statement

The studies involving human participants were reviewed and approved by Ethics committee of the University of Nurnberg-Erlangen. The patients/participants provided their written informed consent to participate in this study.

## Author Contributions

PZ, OM, and VS contributed to conception and design of the study. PZ and MB organized the database. PZ and ME performed the statistical analysis. PZ and FE wrote the first draft of the manuscript. PZ wrote sections of the manuscript. All authors contributed to manuscript revision, read, and approved the submitted version.

## Conflict of Interest

The authors declare that the research was conducted in the absence of any commercial or financial relationships that could be construed as a potential conflict of interest.

## Publisher’s Note

All claims expressed in this article are solely those of the authors and do not necessarily represent those of their affiliated organizations, or those of the publisher, the editors and the reviewers. Any product that may be evaluated in this article, or claim that may be made by its manufacturer, is not guaranteed or endorsed by the publisher.
